# Synergism across neurodegenerative diseases using biological definitions

**DOI:** 10.1002/alz.095055

**Published:** 2025-01-09

**Authors:** Kathleen L. Poston

**Affiliations:** ^1^ Stanford University School of Medicine, Stanford, CA USA

## Abstract

The ability to define neurodegenerative diseases of aging based of biology rather than clinically defined syndromic presentations follows from biomarker development and reflects underlying pathology in living people. The NIA‐AA Research Framework (Jack et al 2018 Alzheimers Dement) proposed the biological definition for Alzheimer’s disease (AD) and sought to provide a common language to identify and stage research participants, who were not cognitively impaired but had abnormal AD biomarkers; it has been revised. The biological classification of Huntington’s disease (Tabrizi et al 2022 Lancet Neurology) included a biological research definition with an integrated staging system, centered on biological, clinical, and functional assessments. Simuni et al (2024 Lancet Neurology) proposed the Neuronal Synuclein Disease (NDS) biological definition, also with integrated staging system, encompassing anyone with biomarker evidence of predominantly neuronal alpha‐synuclein pathology, including people with Parkinson’s disease, dementia with Lewy bodies, or related clinical phenotypes, based on the seed amplification assay biomarker. A key paradigm shift central across these neurodegenerative diseases is diagnosis is not based on the clinical manifestations, but rather disease occurs and can be diagnosed regardless of specific, or in some cases without any, clinical signs or symptoms. It’s recognized these disease processes begin before the onset of symptoms, possibly decades before. Thus, these biological definitions translate this long‐touted recognition into actional research frameworks. There are numerous implications for such a paradigm shift. For one, this allows people to enroll in clinical studies and in clinical trials before the manifestation of classic clinical syndromes, and is likely the only way to test potential therapies for indications that prevent clinical symptoms in people with underlying disease. Further, by separating the syndrome from biology, these definitions recognize that syndromic presentations are not always specific for the underlying pathology, addressing the ongoing challenge of clinical heterogeneity in trials. Another implication of these biological definitions is the co‐occurrence of multiple pathologies, a long under‐recognized entity, can be prospectively studied in living people across the entire clinical spectrum from asymptomatic to severe functional impairment. As these biological definitions iterate upon newly obtainable and validated biomarker data from observational cohorts a more complete molecular characterization of neurodegeneration in aging, independent of syndromic classification, can begin to emerge.

